# ALZHEIMER’S DISEASE RISK REDUCTION HEALTH COACHING: COMPARATIVE ANALYSIS

**DOI:** 10.1093/geroni/igad104.3177

**Published:** 2023-12-21

**Authors:** Faika Zanjani, Brian Battle, Joann Richardson

**Affiliations:** Virginia Commonwealth University, Richmond, Virginia, United States; Virginia Commonwealth University, Richmond, Virginia, United States; Virginia Commonwealth University, Richmond, Virginia, United States

## Abstract

Recent guidelines point to lifestyle change as a tool for decreasing Alzheimer’s disease (AD) risk. To address the limited practice of AD risk reduction and the greater AD prevalence in minority groups, this study aimed to explore the feasibility of a community-level lifestyle intervention targeting high-risk groups. Diverse older adults (60+) living in the Richmond, VA local area, with incomes below $12,000/year and managing diabetes/cardiovascular disease were offered weekly lifestyle telephone-health coaching for 12-weeks in 2019-2020 (Phase 1). The health coaching sessions provided AD lifestyle risk reduction education and goal setting/planning. The intervention sample (n=40, mean age 68 years (range: 60-76 years)) was 90% African American/Black (n=36), 45% males (n=18). Thereafter 2021-2022, n=37 individuals in the same area were recruited as a comparison group and not given health coaching (Phase 2), mean age 65.5 years (range: 57-83 years) was 92% African American/Black (n=34), 50% males (n=18). Demographic differences across groups, indicated that the intervention group was more likely to live alone (p<.01). Repeated measures intervention effects were seen for cognitive ability, indicating greater gains in the intervention group (p<.01). Significant difference score effects indicated greater cognitive ability (p<.01) and physical activity (p<.001) gains in the intervention group; with less physical activity gains (p<.02) for subjective cognitive decline group. This preliminary work creates the impetus for future large-scale AD risk reduction investigations to improve modifiable risk among diverse older adults. Our positive trends in AD risk reduction support the feasibility of telephone-based health coaching AD risk reduction interventions.

